# Identifying bidirectional total and non-linear information flow in functional corticomuscular coupling during a dorsiflexion task: a pilot study

**DOI:** 10.1186/s12984-021-00872-w

**Published:** 2021-05-04

**Authors:** Tie Liang, Qingyu Zhang, Xiaoguang Liu, Bin Dong, Xiuling Liu, Hongrui Wang

**Affiliations:** 1 Institute of Electric Engineering, Yanshan University, Qinhuangdao, 066004 Hebei China; 2 Key Laboratory of Digital Medical Engineering of Hebei Province, Hebei University, Baoding, 071002 China; 3 Development Planning Office, Affiliated Hospital of Hebei University, Baoding, 071002 China

**Keywords:** Functional corticomuscular coupling, Time-delayed maximal information coefficient, Information flow, Nonlinear coupling

## Abstract

**Background:**

The key challenge to constructing functional corticomuscular coupling (FCMC) is to accurately identify the direction and strength of the information flow between scalp electroencephalography (EEG) and surface electromyography (SEMG). Traditional TE and TDMI methods have difficulty in identifying the information interaction for short time series as they tend to rely on long and stable data, so we propose a time-delayed maximal information coefficient (TDMIC) method. With this method, we aim to investigate the directional specificity of bidirectional total and nonlinear information flow on FCMC, and to explore the neural mechanisms underlying motor dysfunction in stroke patients.

**Methods:**

We introduced a time-delayed parameter in the maximal information coefficient to capture the direction of information interaction between two time series. We employed the linear and non-linear system model based on short data to verify the validity of our algorithm. We then used the TDMIC method to study the characteristics of total and nonlinear information flow in FCMC during a dorsiflexion task for healthy controls and stroke patients.

**Results:**

The simulation results showed that the TDMIC method can better detect the direction of information interaction compared with TE and TDMI methods. For healthy controls, the beta band (14–30 Hz) had higher information flow in FCMC than the gamma band (31–45 Hz). Furthermore, the beta-band total and nonlinear information flow in the descending direction (EEG to EMG) was significantly higher than that in the ascending direction (EMG to EEG), whereas in the gamma band the ascending direction had significantly higher information flow than the descending direction. Additionally, we found that the strong bidirectional information flow mainly acted on Cz, C3, CP3, P3 and CPz. Compared to controls, both the beta-and gamma-band bidirectional total and nonlinear information flows of the stroke group were significantly weaker. There is no significant difference in the direction of beta- and gamma-band information flow in stroke group.

**Conclusions:**

The proposed method could effectively identify the information interaction between short time series. According to our experiment, the beta band mainly passes downward motor control information while the gamma band features upward sensory feedback information delivery. Our observation demonstrate that the center and contralateral sensorimotor cortex play a major role in lower limb motor control. The study further demonstrates that brain damage caused by stroke disrupts the bidirectional information interaction between cortex and effector muscles in the sensorimotor system, leading to motor dysfunction.

## Background

In the process of human voluntary movement, the motor cortex of the brain sends out instructions to control muscle actions through the motor nerve pathway, and the sensory information of the muscle is fed back to the cortex through the sensory nerve pathway to ensure the accurate execution of the action [1–3]. This information interaction can be quantified by the coupling relationship between the EEG signal and the SEMG signal of the effector muscle with the development of noninvasive, high-time resolution scalp EEG acquisition technology. Therefore, the functional corticomuscular coupling (FCMC) has become an important way to reveal the control-feedback mechanism of the nervous system and to evaluate the motor function and rehabilitation effect on patients with neurological diseases such as stroke [3–6].

The key challenge to constructing the interrelationship between complex neurophysiological signals is to accurately capture the information flow between the signals. More specifically, it includes two important indicators: direction and strength. Studies were conducted on FCMC from these two aspects. The coherence method is one of the main methods to quantify the functional coupling between the cerebral motor cortex and the effector muscle [7, 8]. However, previous studies confirmed that the information interaction between the motor cortex and the effector muscles was directional [3, 9]. The lack of ability to identify the direction of information interaction limits the application of the coherence method in the analysis of FCMC. Granger causality (GC) and its extension methods used as directional methods to measure the causal relationship between time series have been applied in the analysis of FCMC [2, 3, 10]. The GC method is based on a linear autoregressive model, and its statistical nature is a prediction of stationary time-series data [11]. However, neurophysiological signals have proved to be nonlinear [12–14].Therefore, the effectiveness of GC in analyzing the relationship between nonlinear neurophysiological signals is also questioned [4, 15]. Model-free methods have been used in recent years to analyze the information interaction between neurophysiological signals so as to address the challenge of nonlinearity. The commonly methods are mutual information (MI) and transfer entropy (TE).

The MI method evaluates the interaction relationship between two random variables *X* and *Y* by measuring the shared information between them [16]. MI can detect the linear and nonlinear statistical correlations between two signals, and therefore is widely used in the field of neuroscience [17–19]. However, MI is a symmetrical measurement method that cannot determine the direction of information flow. To solve this problem, Vastano et al. proposed time-delay mutual information (TDMI) to detect information transmission in spatiotemporal systems [20]. TDMI was then introduced into the analysis of information transmission between neurophysiological signals [6, 21, 22]. Nonetheless, it is difficult for both MI and TDMI to accurately estimate the probability density function (PDF) and joint probability density function (JPDF) in the calculation process for short and complex time series [23]. On the other hand, different estimation methods also directly affect the accurate establishment of the relationship between signals.

The TE method is also a model-free method based on information entropy, with the ability to detect linear and nonlinear coupling [24]. Benefited by its asymmetric and transition probability calculation characteristics, TE was considered to be an effective method for detecting causality between neurophysiological signals in recent years [25, 26]. Unfortunately, TE cannot accurately detect the coupling in practical applications when the time series is not long enough [4, 21].

Reshef et al. proposed the maximal information coefficient (MIC) method in 2011 [27]. The generality attribute of MIC meets the requirements of measuring different functional relationships; its equitability attribute ensures that different functional relationships obtain similar measured values at the same noise level. In particular, Reshelf et al. were the first to propose a formula to calculate the nonlinear components of the relationship between two variables, that is, MIC-*ρ*^2^, where *ρ* represents the Pearson correlation coefficient. On the contrary, neither MI nor TE can identify pure nonlinear coupling because the results include both linear and nonlinear coupling. Due to the aforementioned advantages, MIC was widely used in the field of neuroscience [28–30]. In our previous study, MIC was first applied to the analysis of linear and nonlinear coupling components in FCMC [31]. However, limited by the symmetry of mutual information, MIC is also symmetric, that is, MIC(*X*, *Y*) = MIC(*Y*, *X*), so MIC fails to identify the direction of information interaction between signals. To our knowledge, none of the above MIC-based studies analyzed directional specificity in information interaction.

To overcome this limitation, a time-delayed maximal information coefficient (TDMIC) method was proposed in this study by introducing a time-delay parameter to capture the information transmission delay between two short time series. The algorithm was first tested with simulated data to verify the effectiveness of this method. Linear and nonlinear systems with short data lengths were constructed to compare the performance of TDMIC, TDMI, and TE (kernel estimator) in identifying the direction of information flow. As an application of experimental data, the TDMIC method was applied to explore the directional specificity of total and nonlinear information flow of healthy controls and stroke patients in FCMC in a specific frequency band. This study provided a new perspective for exploring the characteristics of FCMC.

## Methods

### Time-delayed maximal information coefficient

For the finite data set *D* of ordered pairs, the data points {*x*; *y*} were distributed in a two-dimensional space, and the data space was divided into *x*-by-*y* grids. In this case, the MI of the two variables was expressed as:

1 $$I(D,{{x}},y) = \sum\limits_{{x \in X}} {\sum\limits_{{y \in Y}} {{{p}}(x,y)\log \left( {\frac{{p(x,y)}}{{p(x)p(y)}}} \right)} }$$

where *p*(*x,* *y*) is the JPDF of time series *X* and *Y*, and *p*(*x*) and *p*(*y*) are the marginal PDF of *X* and *Y*, respectively. PDF and JPDF were obtained by calculating the probability of data points in *D* that fell into each grid.

When the number of grids *x*-by-*y* was fixed, different grid division methods were used. The maximum value of MI among all grid division methods was determined:

2 $$I^{*} (D,{\text{x}},y) = \max (I(D,x,y)$$

To facilitate comparison across grids with different dimensions, $I^{*} (D,{\text{x}},y)$ was normalized by log min{*x*; *y*}, and then the characteristic matrix *M* of a set of data *D* was defined as follows:

3 $$M\left( D \right)_{{\text{x,y}}} = \frac{{I^{*} \left( {D,{\text{x}},y} \right)}}{{\log \min \left\{ {{\text{x}},y} \right\}}}$$

After all elements in the matrix *M* were normalized, the score range obtained was between 0 and 1.

For the data set *D* of ordered pairs with sample size *n*, the MIC was defined as the maximum value of the characteristic matrix obtained by all grid partitioning:

4 $$MIC\left( D \right) = \mathop {\max }\limits_{{xy < B\left( {\text{n}} \right)}} \left\{ {M\left( D \right)_{x,y} } \right\}$$

where the grid size *x*-by-*y* was limited with $B(n)^{{(B(n) = n^{0.6} )}}$ to reduce the calculation efforts. The range of the MIC value was [0, 1]; the higher the score, the stronger the correlation between the two variables.

In addition, RESHEIF et al. defined a natural measure of nonlinearity as follows [27]:

5 $$NL = MIC - \rho^{2}$$

where *ρ* denotes the Pearson product-moment correlation coefficient. When the *NL* value was greater than 0, it indicated a nonlinear relationship.

MIC had the characteristic of symmetry, that is, for variables *X* and *Y*

6 $$MIC\left( {X,Y} \right) = MIC\left( {Y,X} \right)$$

Therefore, MIC could not identify the direction of information flow. In this study, a time-lag parameter was introduced, and the ability to detect information transmission between two signals was obtained by calculating MIC with different time lags (*τ*), which was named TDMIC.

7 $$\begin{gathered} TDMIC = MIC\left( {X,Y,\tau } \right) = \mathop {\max }\limits_{{xy < B\left( {\text{n}} \right)}} \left\{ {\frac{{\max (I_{G} (X,Y,\tau )}}{{\log \min \left\{ {{\text{x}},y} \right\}}}} \right\} \hfill \\ { = }\mathop {\max }\limits_{{xy < B\left( {\text{n}} \right)}} \left\{ {\frac{{\max \left( {\sum\limits_{{x_{t} }} {\sum\limits_{{y_{t - \tau } }} {{\text{p}}(x_{t} ,y_{t - \tau } )\log \left( {\frac{{p(x_{t} ,y_{t - \tau } )}}{{p(x_{t} )p(y_{t - \tau } )}}} \right)} } } \right)}}{{\log \min \left\{ {{\text{x}},y} \right\}}}} \right\} \hfill \\ \hfill \\ \end{gathered}$$

where, *I*_*G*_ (*X,* *Y,* *τ*) is the MI of the time delay in the case of *x*-by-*y* (G). When the information of *X* at time *t* was decomposed at *Y* at time *t* + *τ*, the JPDF between *Y* and *X* had an obvious peak at time *t* + *τ*. Naturally, *I*_*G*_ (*X,* *Y,* *τ*) was larger than *I*_*G*_ (*X,* *Y*). Therefore, the sign of the time lag where MIC(X, Y, τ) reached its peak was used to infer the direction of information flow between *X* and *Y*.

For the experimental application, to estimate the total flow of information between two physiological time series (EEG and EMG), the cumulative information flow within a certain delay *D* was estimated using the following equation [6, 26].

8 $$C_{TDMIC} = \sum\limits_{i = 1}^{D} {TDMIC(k,i)}$$

In this study, the maximum delay *D* was set to 40 data points, and the step size *k* was set to 1 for calculation.

To compare the performance of the algorithm, TDMI and TE methods are also implemented. TDMI method was refered to the code published by Li et al. [21]. TE method with kernel estimator was refered to the code published by Lizier et al. [32]. We also calculated the nonlinear component of TDMIC (NTDMIC) and its cumulative value (*C*_*NTDMIC*_). All algorithms described in this paper were implemented by MATLAB.

### Verification of the TDMIC algorithm

In this study, directed linear and nonlinear systems were constructed separately to verify the ability of the proposed algorithm to identify the direction of information flow. Furthermore, the Henon map was used to verify the ability of the algorithm to detect the coupling strength. Considering the randomization of the initial values of *X* and *Y*, each model was randomly generated 10 times. Subsequently, the algorithm was applied to the study of FCMC while maintaining ankle dorsiflexion. The data length of both simulation and experimental data was set to 1000 to verify the performance of the proposed algorithm in identifying the information flow between short time series.

### Numerical simulation data

#### Unidirectional dynamical system

First, a unidirectional linear dynamic system was constructed using the following model [33]. The calculations showed that a linear information flow existed from time series *Y* to *X*.

9 $$\begin{gathered} x_{t} = 0.6x_{t - 1} + 0.5y_{t - 1}^{{}} + u_{t} \hfill \\ y_{t} = 0.6y_{t - 1} + v_{t} \hfill \\ \end{gathered}$$

Then a unidirectional nonlinear dynamic system was constructed as follows based on the aforementioned unidirectional linear model. A major nonlinear information flow was observed from *Y* to *X*.

10 $$\begin{gathered} x_{t} = 0.6x_{t - 1} + 0.5y_{t - 1}^{2} + u_{t} \hfill \\ y_{t} = 0.6y_{t - 1} + v_{t} \hfill \\ \end{gathered}$$

#### Bidirectional dynamical system

Second, a bidirectional linear dynamic system was constructed using the following model [21]. That is, a bidirectional linear information flow existed between the time series *X* and *Y* generated by the system.

11 $$\begin{gathered} x_{t} = - 0.1y_{t - 1} + u_{t} \hfill \\ y_{t} = - 0.1x_{t - 1} + v_{t} \hfill \\ \end{gathered}$$

A new bidirectional nonlinear dynamic system was constructed as follows based on the aforementioned bidirectional linear model.

12 $$\begin{array}{*{20}c} {x_{t} = - 0.1(y_{t - 1} )^{2} + u_{t} } \\ {y_{t} = - 0.1(x_{t - 1} )^{2} + v_{t} } \\ \end{array}$$

For all models introduced earlier, *u*_*t*_ and *v*_*t*_ represented two independent and identically distributed (i.i.d) standard Gaussian random variables.

#### HENON map

Henon map was used to verify the ability of TDMIC to detect the direction and strength of information flow between time series. Two time series (*X* and *Y*) with unidirectional coupling relationships were generated using the Henon maps. X and *Y* were the driving system and the response system, respectively, that is, information flow from *X* to *Y*:

$$\left\{ \begin{gathered} x_{i + 1} = 1.4 - x_{i}^{2} + 0.3x_{i - 1} \hfill \\ y_{i + 1} = 1.4 - \left( {Ex_{i} + \left( {1 - E} \right)y_{i} } \right)y_{i} + By_{i - 1} \hfill \\ \end{gathered} \right.$$

where *E* is the coupling parameter with an interval of [0, 1], and the coupling strength between two time series could be changed by adjusting the value of *E*.

### Experimental data

Ten subjects (mean age, 59.2 ± 7.0 years; range, 50–68 years; 8 male) with chronic stroke (more than 3 months after onset of stroke) and ten healthy controls (mean age, 58.7 ± 7.2 years; range, 46–67 years; 8 male) without any history of neurological disease were recruited. Patient demographics are shown in Table 1. All the subjects were able to complete the experiment as required.

**Table 1:** Patients demographics

Patient	Age	Gender	Chronicity	Stroke type	Lesion site		Affected leg
1	57	M	25 months	Ischemia	L internal capsule		R
2	66	M	3.5 months	Ischemia	L basal ganglia, Inferior temporal lobe		R
3	50	M	3 months	Ischemia	L internal capsule		R
4	68	M	3 months	Ischemia	R basal ganglia		R
5	61	F	2 months	Ischemia	L frontal lobe, Periventricular		R
6	50	M	18 months	Ischemia	L basal ganglia		R
7	52	M	12 months	Ischemia	L fronto-temporo -parietal		R
8	57	M	13 months	Hemorrhage	L frontal Lobe, Corona radiata		R
9	64	M	4 months	Ischemia	L frontal Lobe, Corona radiata, Centrum Semiovale		R
10	67	F	5 months	Ischemia	R basal ganglia		R

M denotes male; F denotes female; L denotes left; R denotes right

**Fig. 1:**
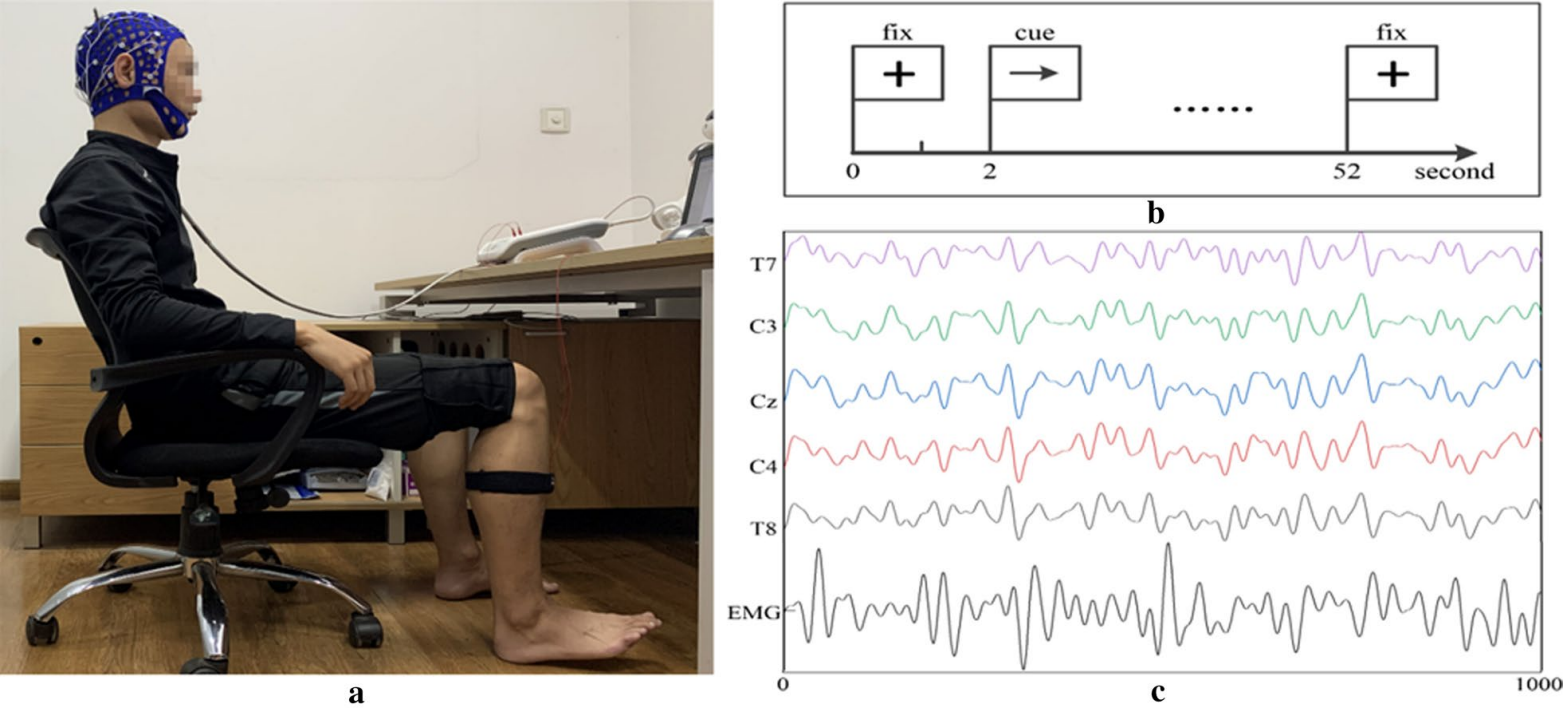
Experimental setup. **a** EEG and EMG acquisition. A participant sat on a chair. EEG and EMG (right TA) signals were simultaneously collected by the amplifier. The participant fixed gaze on the screen in front of them throughout the task and was asked to relax. **b** Experimental paradigm. A cross-shaped mark appeared at the center of the computer screen to remind the participants to pay attention. After 2 s, a right arrow appeared, prompting the participant to dorsiflexion of the right ankle and maintain this state for 50 s. **c** Denoised EEG and EMG signals

The preparation before the experiment was similar to our previous study [31]. The difference is that we changed the experimental paradigm from the autonomic dynamic dorsiflexion task to a steady-state dorsiflexion task to obtain stable data. There was a cross-shaped mark in the center of the computer screen to attract the attention of the participants. After 2 s, a right arrow appeared, prompting the participants to dorsiflexion of the right ankle and maintain this state for 50 s. Figure 1 shows the experimental setup. The participants then rested for 60 s to avoid muscle fatigue. Each participant repeated the aforementioned task five times.

During the study, EEG and EMG signals were simultaneously acquired with an EEG amplifier system (Neuroscan, Australia). Using the international 10–20 system, 26 electrodes were used to record the EEG data (i.e., FP1, FP2, Fz, F3, F4, F7, F8, FC3, FCz, FC4, C3, Cz, C4, CP3, CPz, CP4, P3, Pz, P4, T7, T8, P7, P8, O1, Oz, and O2). The EMG signal from the tibialis anterior (TA) of the right leg was recorded with bipolar electrodes. EEG and EMG data were sampled at 1024 Hz. The electrode wires were fixed with a tape to reduce motion artifacts caused by shaking. The data were maintained for 2–49 s for subsequent analysis to obtain the data under the steady state. Finally, 5 48 s-long epochs free of artifacts in each participant were obtained. Data were further cut into 1000 data point segments with no overlapping. 50 Hz power frequency interference was removed, and Band-pass filtering (2–100 Hz) was performed on EEG. Then, the independent component analysis (ICA) algorithm was used to remove artifacts, such as electrooculogram (EOG) and EMG. For EMG, a notch filter was used to remove the 50 Hz power frequency interference, and a 2-stage IIR bandpass filter (5–100 Hz) was performed to remove low-frequency noise.

TDMIC and NTDMIC (the nonlinear component of TDMIC) values were calculated from the beta (14–30 Hz) and gamma (31–45 Hz) bands in the power temporal power map of EEG and EMG obtained by Morlet wavelet transform. Cumulative values of TDMIC and NTDMIC (i.e.C_TDMIC_ and C_NTDMIC,_ represents the total information flow and the nonlinear information flow) were calculated for 40 data point delay. The estimated time delay was close to 40 ms, which was in the range of latencies (20–40 ms) reported between the cortex and muscles [3, 9, 34, 35].

### Statistical significance

In this study, the permutation test was used for significance testing. The two original time series were randomly shuffled to generate surrogate data. As for simulated data, the significance level alpha was set to 0.01. For experimental data, the repeated-measures analysis of variance (rANOVA, *a* = 0.05) was performed on TE, TDMI, and TDMIC. Greenhouse–Geisser correction was used to correct the degree of freedom. Bonferroni correction was used for multiple comparisons. All statistical analyses were conducted in SPSS/PC, version 22.0 (SPSS Inc., IL, USA).

## Results

### Results for numerical models

**Fig. 2:**
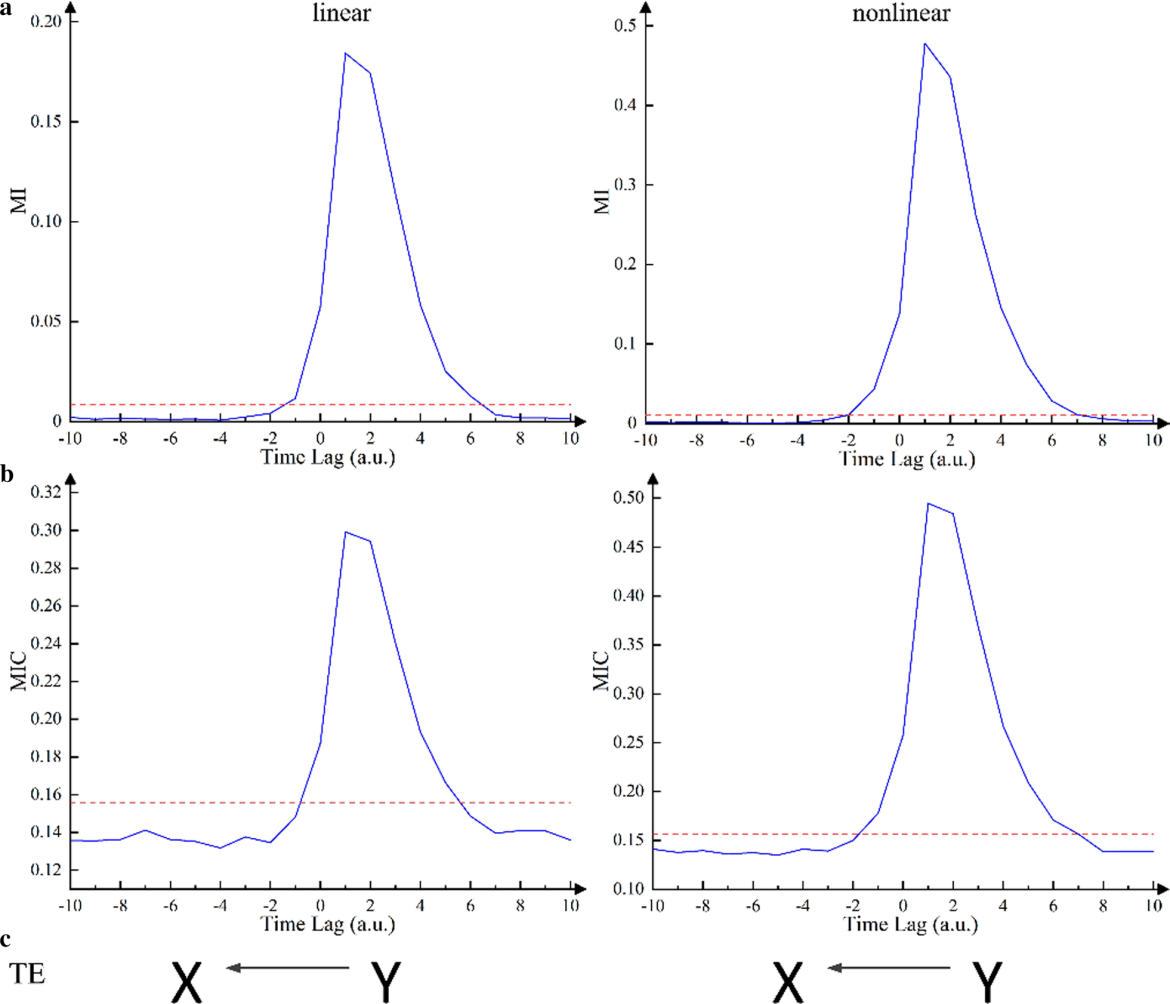
**a** TDMI and **b** TDMIC curves as a function of time lag. **c** Causal direction identified by TE. The left and right columns correspond to the unidirectional linear and nonlinear dynamical systems, respectively. X-axis indicates delay time with arbitrary unit (a.u.). Red dotted lines are the levels of significance threshold. The data length of all models was set to 1000

Figure 2 indicates TDMI and TDMIC values as a function of time lag from two time series generated from the unidirectional models. As shown in Fig. 2, whether it was a linear system or a nonlinear system, both the TDMI and TDMIC curves reached a significant large peak at the positive time lag (linear: *τ* = 1, nonlinear: *τ* = 3). The peak values were significantly greater than the significance threshold (*a* = 0.01). This finding indicated that the direction of information flow recognized by TDMI and TDMIC for unidirectional linear and nonlinear systems was *Y* to *X*, which was consistent with the information flow direction of unidirectional models. The TE analysis on the linear system showed that the TE value from *X* to *Y* was 3.84 × 10^–2^ and that from *Y* to *X* was 2.399 × 10^–1^. The significance threshold of TE obtained by the permutation test was 9.53 × 10^–2^. This finding indicated that the TE method recognized unidirectional information flow consistent with the model, that is, from *Y* to *X*. On the contrary, for the nonlinear system model, the TE value from *X* to *Y* was 3.48 × 10^–2^ and that from *Y* to *X* was 1.104 × 10^–1^. Both the TE values were above the significance threshold. Hence, the TE method was able to identify the causal relationship between *X* and *Y* that the model suggested. The results of TE are summarized in Table 2.

**Table 2:** Summary of te results in each model

Model	Threshold TE_x→y_	Threshold TE_y→x_	TE_x→y_	TE_y→x_	TE direction
Unidirectional linear dynamic system (X ← Y)	9.53 × 10^–2^	9.93 × 10^–2^	3.84 × 10^–2^	2.399 × 10^–1^	X ← Y
Unidirectional nonlinear dynamic system (X ← Y)	1.049 × 10^–1^	1.109 × 10^–1^	3.48 × 10^–2^	1.104 × 10^–1^	X ← Y
Bidirectional linear dynamic system (X $\leftrightarrow$ Y)	4.75 × 10^–2^	4.78 × 10^–2^	3.65 × 10^–2^	4.03 × 10^–2^	Independent
Bidirectional nonlinear dynamic system (X $\leftrightarrow$ Y)	4.95 × 10^–2^	4.67 × 10^–2^	3.55 × 10^–2^	3.45 × 10^–2^	Independent
Henon Map (X → Y)	5.37 × 10^–2^	5.21 × 10^–2^	7.195 × 10^–1^	8.99 × 10^–2^	X $\leftrightarrow$ Y

**Fig. 3:**
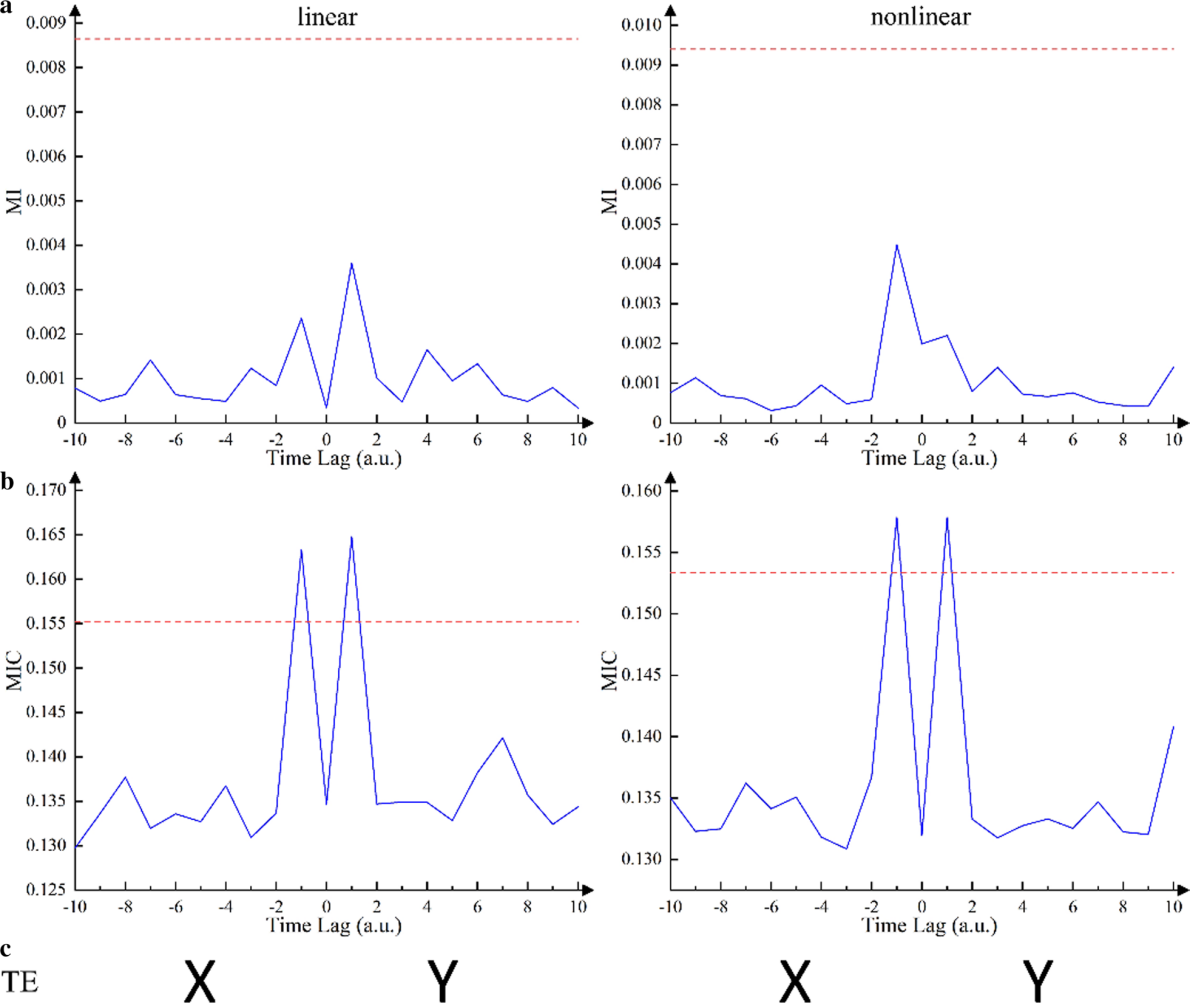
**a** TDMI and **b** TDMIC curves as a function of time lag. **c** Causal direction identified by TE. The left and right columns correspond to the bidirectional linear and nonlinear dynamical systems, respectively. X-axis indicates delay time with arbitrary unit (a.u.). Red dotted lines are the levels of significance threshold. The data length of all models was set to 1000

Figure 3 indicates TDMI and TDMIC values as a function of time lag from two time series generated from the bidirectional models. Two obvious peaks were observed in TDMIC curves, as shown in Fig. 3(b), which were located at positive and negative lags (linear: *τ* =  ± 1, nonlinear: *τ* =  ± 1). Both peaks were significantly greater than the significance threshold level (a = 0.01). This finding indicated that the bidirectional information flow between *X* and *Y* was detected by TDMIC, which was consistent with the models. On the contrary, as shown in Fig. 3a, no obvious peaks above the significance threshold were observed in the TDMI curves. This observation showed that TDMI failed to identify the direction of the information flow of bidirectional systems under this data length (1000). As shown in Table 2, whether it was a bidirectional linear or nonlinear model, both the TE values were below the significance threshold, indicating that the TE method did not recognize a significant information flow between *X* and *Y*. This was also inconsistent with the models.

**Fig. 4:**
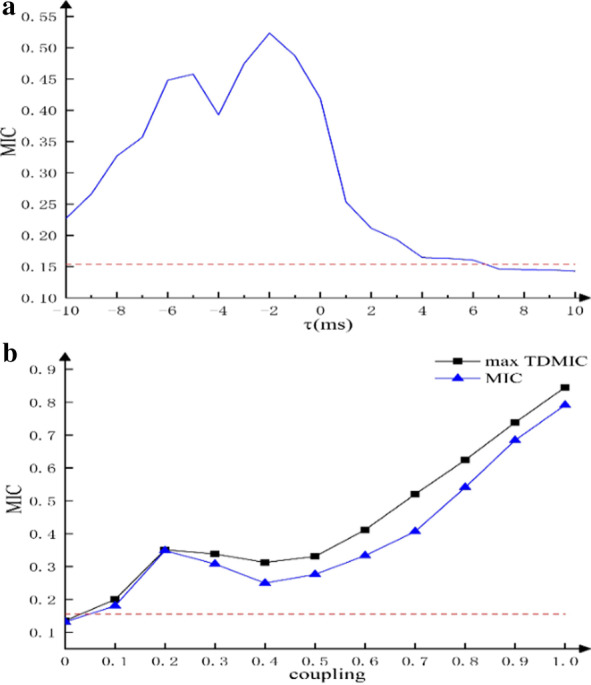
**a** TDMIC curves as a function of time lag in Henon map (*E* = 0.7). X-axis indicates delay time with arbitrary unit (a.u.). **b** TDMIC detected the coupling strength with the variation of parameters (E). Here Henon map was set to a nonidentical (*B* = 0.1) system with unidirectional coupling (*X* to *Y*). The data length was set to 1000

As shown in Fig. 4a, a peak value greater than the significance threshold was observed at the negative lag (*τ* = –1). This indicated that the direction of information flow recognized by TDMIC for the Henon map was *X* to *Y*, which was consistent with the model. In contrast, the information flow direction of the unidirectional Henon map was misinterpreted as bidirectional by TE, as shown in Table 2. Figure 4b shows the ability of TDMIC to detect the coupling strength following the change in the coupling parameter *E*. The maximum value of TDMIC values also increased monotonically with *E*. Additionally, a local maximum was observed in Fig. 4b around *E* = 0.2.

## Results for experimental data

**Fig. 5:**
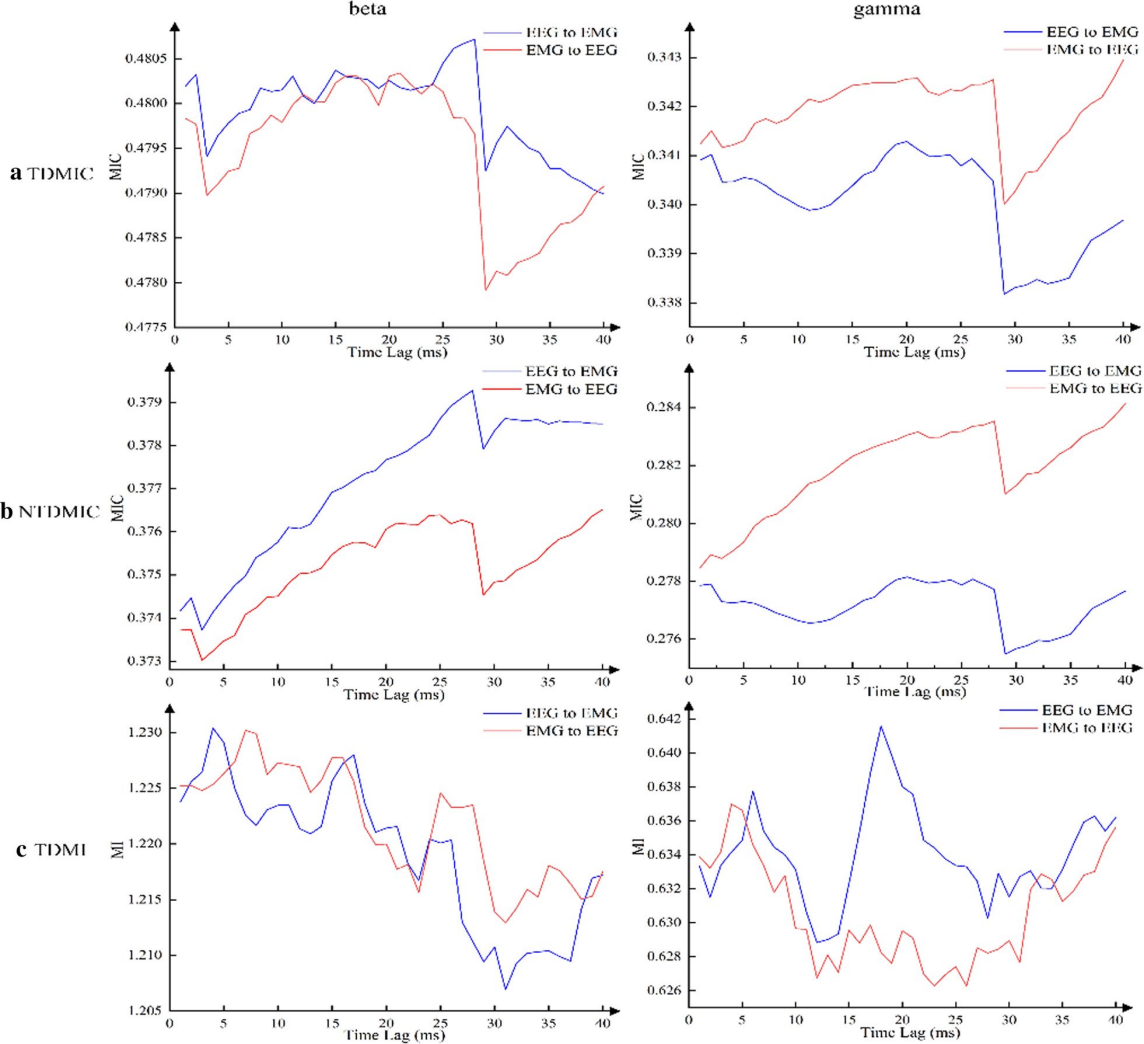
Grand averaged **a** TDMIC, **b** NTDMIC, and **c** TDMI curve between EEG (Cz) and EMG as a function of delay data points in the beta (14–30 Hz) and gamma (31–45 Hz) bands for controls

Figure 5a and b presents the grand averaged TDMIC and NTDMIC curves as a function of delay in the beta (14–30 Hz) and gamma (31–45 Hz) bands. The EEG signal collected at the Cz position was selected for analysis. Overall, whether it was the TDMIC or the NTDMIC, the beta-band information flow from EEG to EMG was stronger than that from EMG to EEG. Interestingly, the ascending information flow (EMG to EEG) in the gamma band was higher than the descending information flow (EEG to EMG). Figure 5c presents the grand averaged TDMI curve as a function of delay in the beta and gamma bands. Compared with TDMIC and NTDMIC, the TDMI curve did not clearly distinguish the ascending and descending information flows. The interval of significance thresholds for TDMIC, NTDMIC and TDMI in the beta band were [0.1546, 0.1554], [0.152, 0.1528] and [0.0030, 0.0035]. In the gamma band, the interval of significance thresholds for TDMIC, NTDMIC and TDMI were [0.1460, 0.1464], [0.1430, 0.1439] and [0.0032, 0.0035], respectively. Both of the TDMIC and NTDMIC values were above the significance thresholds. However, TDMI was below the significance threshold. Additionally, the TE method did not detect significant bidirectional information flow between experimental data. The TE results of the experimental data are listed in Table 3.

**Table 3:** Summary of te results on experimental data

Frequency band	EEG → EMG	EMG → EEG	Threshold TE_EEG→EMG_	Threshold TE_EMG→EEG_
Beta (14–30 Hz)	0.0072	0.0079	0.0584	0.0594
Gamma (31–45 Hz)	0.0093	0.0098	0.0634	0.0651

**Fig. 6:**
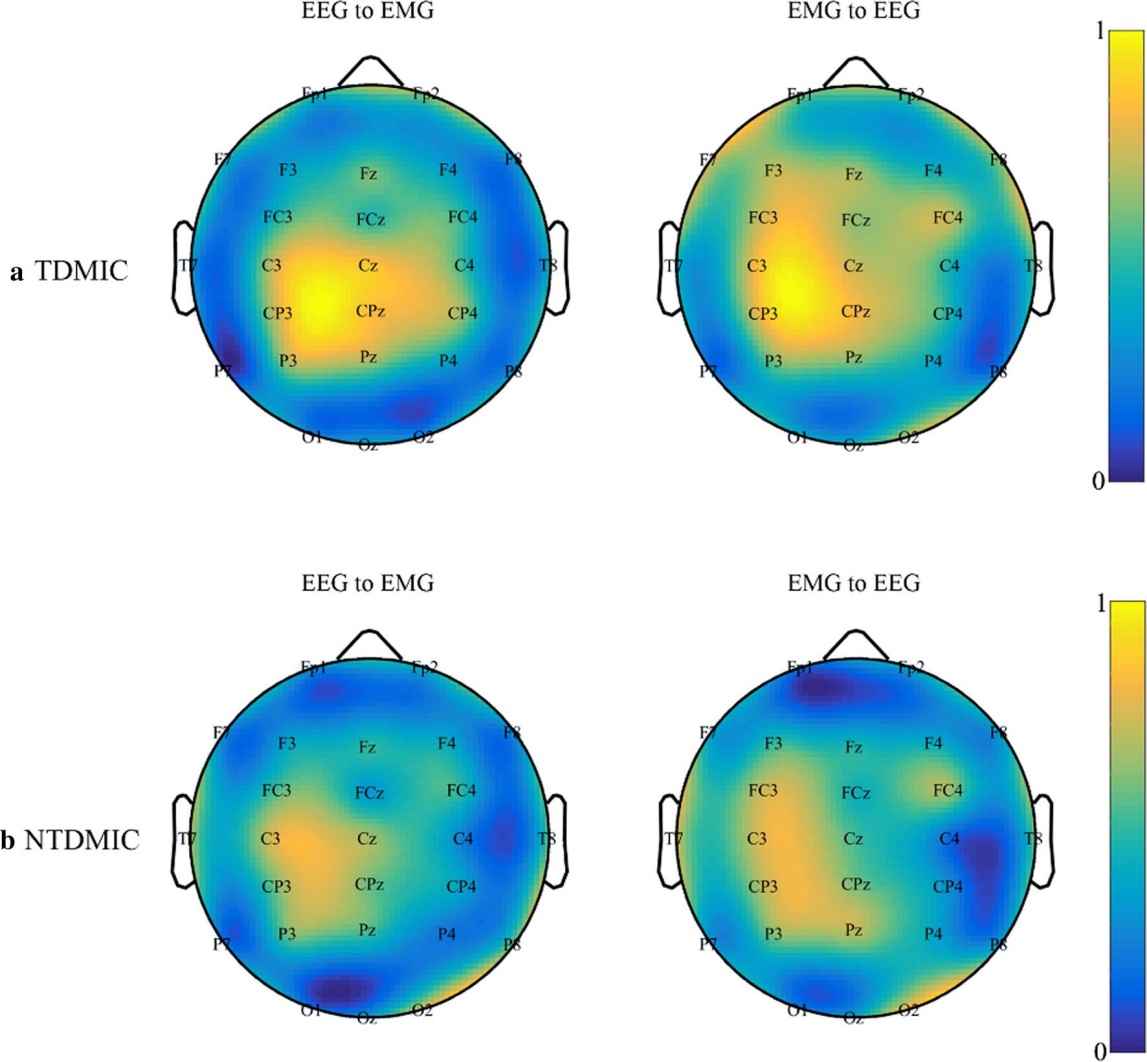
Grand averaged normalized topographies of C_TDMIC_ (**a**) and C_NTDMIC_ (**b**) in the beta band of controls for EEG → EMG (left column) and EMG → EEG (right column) directions

Figure 6 shows the grand averaged normalized topographies of C_TDMIC_ and C_NTDMIC_ for controls. The averaged C_TDMIC_ topography of EMG → EEG was similar to that of EEG → EMG with the peak value at similar electrodes: Cz, C3, CP3, P3, Pz and CPz. The difference was that the peak area of the EMG → EEG topographic map was more scattered. In addition, the peak distribution of C_NTDMIC_ in the two directions (EMG → EEG and EEG → EMG) was similar to that of C_TDMIC_, which was mainly distributed at Cz, C3, CP3, P3, Pz and CPz.

**Fig. 7:**
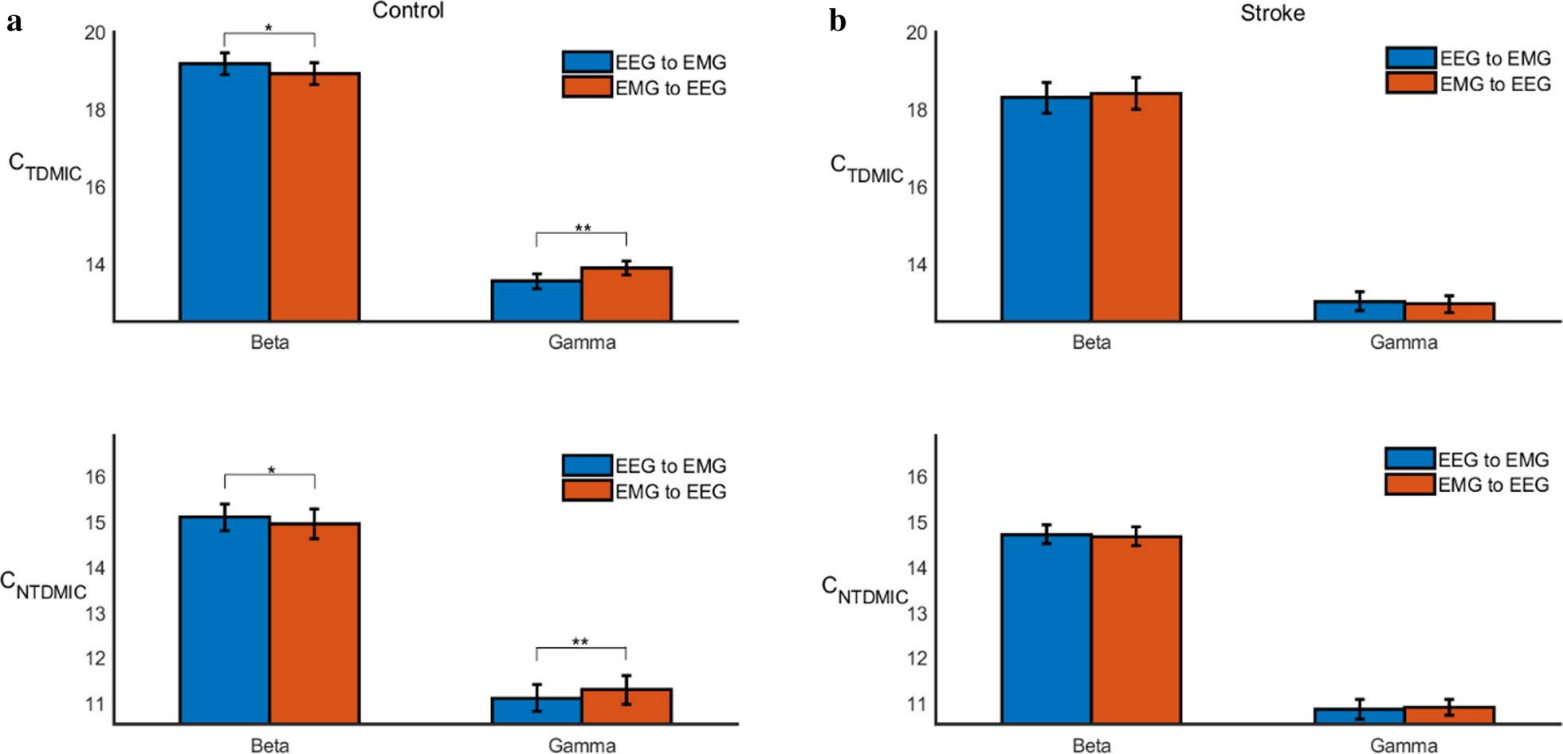
Grand averages of the C_TDMIC_ and C_NTDMIC_ values in both directions (i.e., EEG to EMG, EMG to EEG) for all subjects at beta and gamma bands. “*” denotes P < 0.05, “**” denotes P < 0.01, and “***” denotes P < 0.001

The cumulative values of TDMIC and NTDMIC in the beta and gamma bands for both directions were calculated to further quantify the differences between the controls and the stroke patients. Then, three way rANOVA was performed for each method, with subject (two levels: stroke and healthy control) as a between-subject factor, with frequency band (two levels: beta and gamma) and direction (two levels: descending and ascending) as within-subject factors. Figure 7a shows the results of statistical analysis for controls. The results showed that the C_TDMIC_ and C_NTDMIC_ in the beta band were significantly higher in the descending direction than in the ascending direction (C_TDMIC_: F(1, 18) = 5.07, *p* = 0.037; C_NTDMIC_: F(1, 18) = 7.86, *p* = 0.012). On the contrary, the ascending C_TDMIC_ and C_NTDMIC_ in the gamma band were significantly higher than the descending values (C_TDMIC_: F(1, 9) = 8.56, *p* = 0.009; C_NTDMIC_: F(1, 9) = 11.18, *p* = 0.004). Figure 7 (b) shows the results of statistical analysis for stroke groups. Different from the control groups, the C_TDMIC_ and C_NTDMIC_ results showed no significant difference in beta or gamma bands in both directions (beta band, C_TDMIC_: F(1, 18) = 4.20, *p* = 0.055; beta band, C_NTDMIC_: F(1, 18) = 0.54, *p* = 0.473; gamma band, C_TDMIC_: F(1, 18) = 2.19, *p* = 0.156; gamma band, C_NTDMIC_: F(1, 18) = 0.76, *p* = 0.396).

**Fig. 8:**
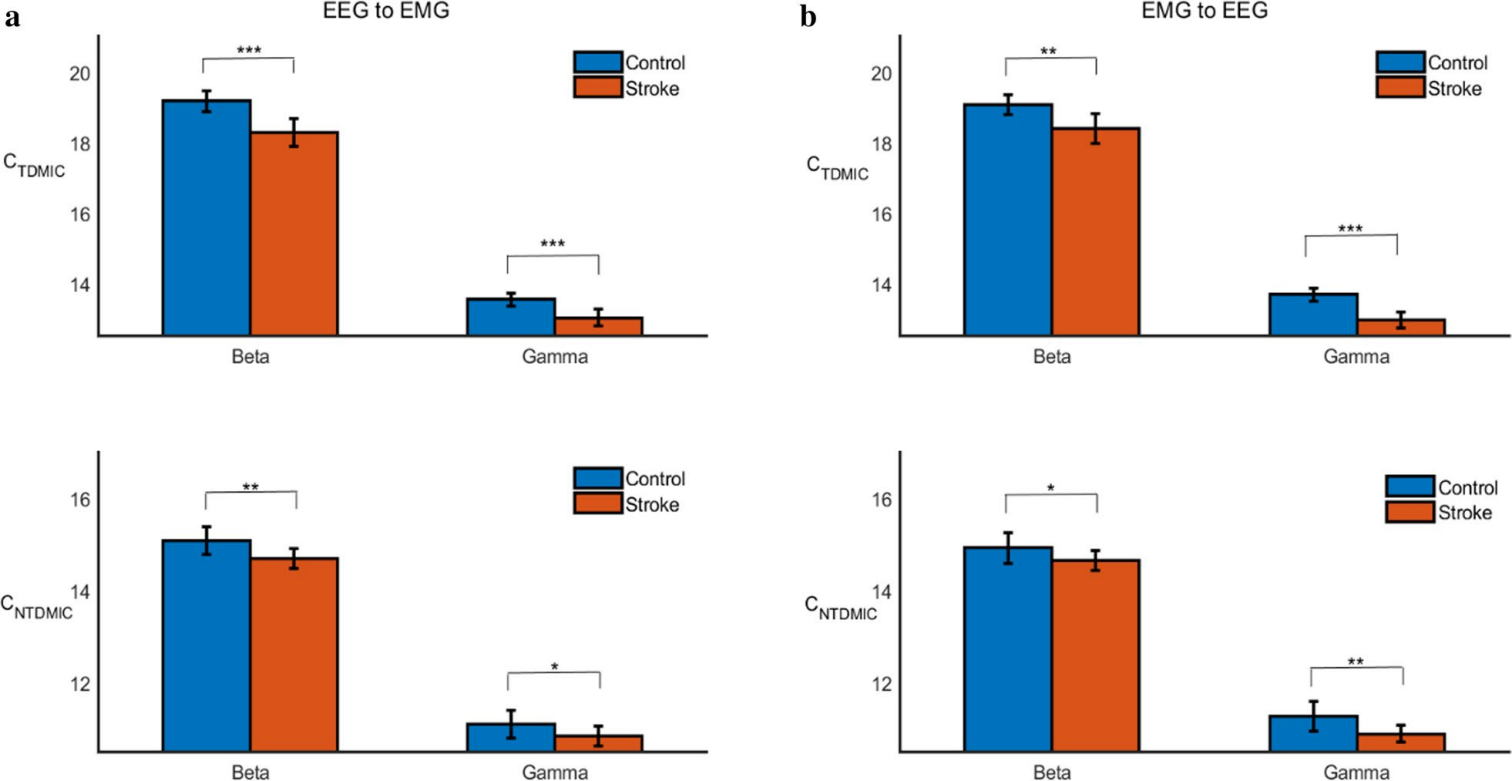
Comparison of the C_TDMIC_ and C_NTDMIC_ values betwen controls and stroke patients in both directions (i.e., EEG to EMG, EMG to EEG) at beta and gamma bands, respectively. “*” denotes *p* < 0.05, “**” denotes *p* < 0.01, and “***” denotes *p* < 0.001

Furthermore, compared to the controls, the C_TDMIC_ and C_NTDMIC_ results of the stroke groups were significantly weaker both in the beta and gamma bands in the descending direction (i.e., EEG to EMG), as shown in Fig. 8a (C_TDMIC_, beta band: F(1,18) = 33.0, *p* = 0.000, Bonferroni; C_TDMIC_, gamma band: F(1,18) = 27.84, *p* = 0.000, Bonferroni; C_NTDMIC_, beta band: F(1,18) = 11.65, *p* = 0.003, Bonferroni; C_NTDMIC_, gamma band: F(1,18) = 4.63, *p* = 0.045, Bonferroni). Similarly, compared to the controls, the results of the stroke groups were significantly weaker both in the beta and gamma bands in the ascending direction (i.e., EMG to EEG), as shown in Fig. 8b (C_TDMIC_, beta band: F(1,18) = 17.46, *p* = 0.001, Bonferroni; C_TDMIC_, gamma band: F(1,18) = 65.68, *p* = 0.000, Bonferroni; C_NTDMIC_, beta band: F(1,18) = 4.81, *p* = 0.042, Bonferroni; C_NTDMIC_, gamma band: F(1,18) = 10.82, *p* = 0.004, Bonferroni).

## Discussion

This study proposed the TDMIC algorithm to solve the problem of inability to identify causal interactions in MIC applications. The simulation results showed that TDMIC could accurately identify the information flow direction of all models with short data lengths and detect the coupling strength of nonlinear systems. On the contrary, with the same short data length, the performance of TE or TDMI was not as good as that of TDMIC in identifying the direction of information flow. The application of experimental data showed significant bidirectional total and nonlinear information flows in FCMC in the beta and gamma bands. Further analysis showed that the strength of total and nonlinear information flow in the descending direction were significantly higher than that in the ascending direction in the beta band, while an opposite phenomenon was observed in the gamma band. Additionally, strong total and nonlinear information flow mainly acted on the center and contralateral sensorimotor cortex. Further controlled experiments showed that the total and nonlinear information flows in both beta and gamma bands were significantly weaker in stroke group than in healthy control group. This study extended the application of MIC and suggested a new idea for the study of nonlinear coupling components in FCMC.

Compared with TE and TDMI, the TDMIC method could more effectively identify the direction of information flow between short time series, which might be related to the derivation of these algorithms. TE was proposed to explore whether the historical information of the driver could improve the prediction of the state of the recipient [24]. The value of TE (*Y* to *X*) between time series *X* and *Y* was expressed by the following formula

14 $$T_{Y \to X} = \sum {p(x_{n + 1} ,x_{n}^{ - } ,y_{n}^{ - } )} \log \frac{{p(x_{n + 1} ,x_{n}^{ - } ,y_{n}^{ - } )}}{{p(x_{n + 1} |x_{n}^{ - } )}}$$

where $x_{n}^{k} = \{ x_{n - 1} ,x_{n - 2} , \ldots x_{n - k} \}$ and $y_{n}^{l} = \{ y_{n - 1} ,y_{n - 2} , \ldots y_{n - l} \}$ are *k*- and *l*-dimensional delay vectors, which represent the history of *X* and *Y*. The formula showed that TE involved the calculation of high-dimensional PDF. This meant that the calculation of TE required long and stable data to accurately construct a high-dimensional PDF [21]. In addition, TE was equivalent to GC under Gaussian conditions [36]. Both GC and TE might detect false causality due to incomplete observation of the state of the drive system [11, 37]. In the case of short data length (1000) in this study, the performance of the TE method was not satisfactory, especially for bidirectional linear and nonlinear systems.

As an asymmetric extension of the MI method based on information theory, TDMI also involved the calculation of the PDF. The accuracy of PDF calculations directly affected the validity of TDMI results. Unlike TE, the dimension of the TDMI PDF was only 2, which avoided the problem of high-dimensional PDF construction in the TE method. Nevertheless, TDMI still needed long stationary time series data to accurately calculate PDF [6]. Roulston et al. used the standard error formula to prove that MI had obvious errors in the case of short data [38]. In this study, TDMI failed to detect the information flow direction on the bidirectional linear and nonlinear models, thus limiting the application of TDMI to nonstationary EEG signals. The brain proved to be a nonlinear dynamic system [13, 14]. It was difficult to obtain long stationary EEG data in motor task experiments. For instance, in this study, the duration of ankle dorsiflexion was about 1 s. The EEG signal was nonstationary and dynamic during the whole action. Therefore, the application of the TDMI method in short-term exercise task experiments needed to be carefully evaluated.

Unlike TE and TDMI, TDMIC was an asymmetric extension based on the MIC algorithm. The MIC algorithm ensured that different types of functional relationships were accurately captured by finding the grid division method with the largest MI value [27]. This was different from TDMI in terms of relying on a single PDF calculation method to calculate MI. Especially for complex time series, a single discrete method was not always suitable for different types of functional relations. MIC solved this problem well using the calculation principle. At the same short data length (1000), the performance of TDMIC in identifying the direction of information flow between time series pairs generated by four different models was significantly better than that of TE and TDMI.

The ability to accurately capture the coupling strength between time series is important for evaluating the effectiveness of a new algorithm. The Henon map results showed that the maximum value of TDMIC increased monotonically as coupling strength increase, which was consistent with the trend of the MIC curve. The local maximum we observed in the result was related to the characteristics of the Henon map, namely, it can be interpreted as the minima of the largest sub-Lyapunov exponent [39]. This was also consistent with the previous studies that used Henon map to verify new algorithms [40–42]. The value of TDMIC was always greater than the value of MIC. The Henon map had a typical nonlinear unidirectional information flow(*X* to *Y*). Therefore, according to the principle of the algorithm, the value of MIC at the time of negative lag was naturally greater than the value at the time lag *τ* = 0. These results indicated that the TDMIC algorithm could accurately identify the coupling strength between nonlinear dynamic systems.

The direction and strength of the information flow in FCMC needed to be accurately identified to evaluate the motor function and reveal the motion control-feedback mechanism. Beta- and gamma-band FCMCs were demonstrated to be associated with movement tasks [8, 43]. The Cz electrode position was considered to be related to leg movement, and therefore EEG signals recorded from the Cz channel were selected for analysis in this study. Significant beta-band total information flow was observed in both descending and ascending directions. This observation was consistent with previous findings indicating not only descending motor output information but also ascending somatosensory feedback information [3, 15, 44]. As the cortex and the periphery constituted a closed-loop sensorimotor system, the interaction between EEG and EMG was inevitably affected by the bidirectional information flow. Further statistical significance showed that the beta-band total information flow in the descending direction was significantly higher than that in the ascending direction, which was consistent with previous findings on the steady-state force output task for the upper limbs [2, 6, 45]. This might be associated with the experimental paradigm of steady-state force output. During the steady-state force output, the task for the upper or lower limbs needed stronger motor control signals than sensory feedback integration. Beta-band oscillations affected the transmission of descending control instructions, which were used for force stability and output [1, 46]. Also, a significant bidirectional gamma-band total information flow was observed. The difference was that the gamma-band information flow in the ascending direction was stronger than that in the opposite direction. This result indicated that the transmission of sensory feedback was the main information flow in the gamma band. The gamma-band coupling was confirmed to be related to the generation of dynamic force and the integration of information such as attention, vision, and proprioception [43, 46]. The stronger somatosensory feedback flow in the gamma band might provide evidence for these conclusions.

Additionally, significant bidirectional nonlinear information flow was observed in beta and gamma bands, which might be accounted for by the mechanism of neural signal production. Motor output and somatosensory feedback were mainly produced by nonlinear neuronal interaction in the cortex [47]. Therefore, the bidirectional information flow in FCMC naturally had obvious nonlinear characteristics. Similar to the total information flow, the direction specificity of the nonlinear information flow might also be caused by the experimental paradigm and the different functions of beta- and gamma-band oscillations. A comprehensive assessment of nonlinear interactions in the sensorimotor system was demonstrated to have clinical significance [48].Our future studies will explore the clinical significance of nonlinear information flow in FCMC.

The coherence between the contralateral sensorimotor cortex and effector muscle (TA) in lower limb tasks has been confirmed by several previous studies [8, 10]. Our study were partially consistent with these previous findings. As shown in the beta-band topographic maps, the strong ascending total information mainly flowed to C3, Cz, CP3, P3 and CPz, from where the descending information was output. These electrode positions are generally thought to be associated with the central and contralateral sensorimotor cortex. What differentiates our study from the previous ones is that we observed strong bidirectional total information flows that mainly act on this region. This finding indicated that the central and contralateral positions of the sensorimotor cortex played a major role in motor control and sensory feedback in lower limb motor tasks. A near-infrared study on gait also confirmed that the medial primary sensorimotor cortices were activated during foot movements [49]. Additionally, the peaks of the topographic map of the total information flow were scattered more in the ascending direction (EMG to EEG) than in the opposite direction (EEG to EMG). This might have to do with the physiological structural difference between the motor control pathway and the sensory feedback pathway. The descending motor output was mainly completed through the corticospinal tract, with direct information transmission. However, the ascending sensory feedback pathway involved the cerebellum, brainstem, and thalamus, with a more complicated information transmission process. The inconsistency in information transmission, which resulted in the positive and negative directions, also showed nonlinear FCMC.

Furthermore, for healthy controls, after separating the nonlinear information flow form FCMC, the bidirectional nonlinear information flow also mainly acted at C3, CP3, P3 and CPz. This was similar to the results of some recent studies on hand tasks [6, 35]. Jin et al. used TDMI to observe a significant nonlinear information flow from the contralateral sensorimotor cortex to the effector muscle during a wrist extension task [6]. However, they did not present a further discussion on the information flow from the effector muscles to the sensorimotor cortex. Recently, Yang et al. used the MSPC method and found the peak of the ascending and descending nonlinear coherences at the CCP3 and C1 electrodes, respectively, during constant contraction of the right upper limb [35]. Our findings indicated that the nonlinear information flow of the contralateral sensorimotor cortex had the dominant role in motor control and sensory feedback regardless of upper or lower limb tasks.

Compared to the healthy controls, we did not observe significant directional differences in the strength of information flow both in the beta and gamma bands. This may be due to structural damage to the patient's brain, which affects normal information interaction [50]. Another possible explanation is that the individual differences among patients caused by factors such as different disease levels, different brain damage locations, and different stroke onset times. Furthermore, compared with the controls, the bidirectional total and nonlinear information flows in both the beta and gamma bands of the stroke group were significantly reduced. This result was consistent with the previous studies [5, 51–53]. This weakening of FCMC may be caused by cortical damage or muscle changes resulting from stroke [5, 54]. On the one hand, neural activities through the pyramidal tract were significantly reduced after brain injury, leading to the disassociation of presynaptic and postsynaptic activities, thereby weakening the cortical-spinal connection [52]. On the other hand, it has been demonstrated that neuromuscular disorders lead to an increased MU discharge variability and a decreased firing rate after stroke [55]. In particular, Mima et al. previously demonstrated that weak coupling is primarily caused by impaired information flow from the brain to the muscles [56]. The results of weaker descending information flow that we observed in the stroke group was the same. Meanwhile, weaker information flow in the ascending direction was also observed in the stroke group. As mentioned earlier, information flow in the ascending direction plays an important role in somatosensory tasks. Weakening of the ascending information flow may have caused the proprioception disorder of the stroke patient who, in fact, usually suffers proprioceptive dysfunction [57].This study may have demonstrate that the cerebral lesion caused by stroke damages the bidirectional information interaction between the cortex and the effector muscles in the sensory-motor system, and this damage leads to obstacles in limb movement control and proprioceptive feedback.

## Conclusions

This study proposed the TDMIC algorithm to address the challenge of accurate identification of information flow in FCMC. Simulation and experimental results showed the effectiveness of the proposed method. This study extended the related research of FCMC on information flow and further explored the frequency specificity and directional specificity of bidirectional nonlinear information flow. The weakening of bidirectional information flow may reflect the underlying mechanism of limb sensorimotor dysfunction after stroke. The proposed method might provide a deeper understanding of the control-feedback mechanism in motor control and serve a useful tool for the clinical evaluation of motor function. Further studies will recruit more stroke patients for a long-term analysis, focusing on evaluating the effects of different rehabilitation strategies on rehabilitation outcomes.

## Data Availability

The datasets of the experiments in the current study are available from the first author on request.

## Abbreviations

FCMC: Functional corticomuscular coupling
EEG: Electroencephalography
SEMG: Surface electromyography
TDMIC: Time-delayed maximal information coefficient
TE: Transfer entropy
MI: Mutual information
GC: Granger causality
TDMI: Time-delay mutual information
PDF: Probability density function
JPDF: Joint probability density function
MIC: Maximal information coefficient
NTDMIC: Nonlinear component of TDMIC
C_TDMIC_ and C_NTDMIC_: Cumulative value of TDMIC and NTDMIC
rANOVA: Repeated-measures analysis of variance
TA: Tibialis anterior
ICA: Independent component analysis
